# Editorial: Exploring cancer stem cells signaling pathways

**DOI:** 10.3389/fonc.2023.1274509

**Published:** 2023-08-18

**Authors:** Alpha R. Mekapogu, Cecilia A. Suárez, Jenny Y. Wang

**Affiliations:** ^1^ Cancer and Stem Cell Laboratory, School of Medical Sciences, Faculty of Medicine and Health, The University of Sydney, Kolling Institute, Sydney, NSW, Australia; ^2^ Laboratorio de Sistemas Complejos, Instituto de Física Interdisciplinaria y Aplicada (INFINA), Consejo Nacional de Investigaciones Científicas y Técnicas (CONICET) – Universidad de Buenos Aires, Buenos Aires, Argentina; ^3^ Departamento de Física, Facultad de Ciencias Exactas y Naturales, Universidad de Buenos Aires, Buenos Aires, Argentina

**Keywords:** cancer stem cell (CSC), signaling/signaling pathways, Wnt/b-catenin, non-canonical Wnt pathway, zinc finger proteins, glycosylation, epithelial mesenchymal transition (EMT), microRNAs (miRNAs)

Cancer stem cells (CSCs) have gained prominence as a critical driver in tumor initiation, progression, and therapeutic resistance (1). Their unique properties of self-renewal and differentiation give rise to differentiated cancer cells making up the bulk of the tumor contributing to tumor heterogeneity (2). The presence of CSCs is clinically associated with poor prognosis and an increased risk of tumor recurrence (3). These cells are mediated by complex pathway events, such as canonical (β-catenin-dependent) and non-canonical (β-catenin-independent) Wnt signaling, which are activated in a context-dependent manner (4). Understanding key oncogenic events controlling CSCs aids in deciphering the mechanisms that drive tumor progression and metastasis, addressing resistance to therapy, exploring tumor heterogeneity, identifying novel therapeutic targets, developing personalized medicine, and discovering diagnostic biomarkers (5, 6). Such efforts are crucial for advancing knowledge in cancer biology and creating more effective strategies for cancer prevention, diagnosis and treatment.

This Research Topic, comprising both original research articles and reviews, investigates oncogenic events in CSCs that govern malignant progression and cancer recurrence, offering insights into the development of new therapeutic strategies to overcome resistance.

The canonical Wnt/β-catenin signaling pathway, for instance, maintains the stemness in breast cancer by activating expression of genes associated with the regulation of CSCs (7). Dysregulation of this pathway can trigger breast cancer development, metastasis, and chemoresistance (4). Additionally, this pathway influences key processes involved in tumorigenesis, including cell proliferation, survival and invasion. A study by Barnawi et al. examines the role for the fascin-focal adhesion kinase (FAK)-β-catenin axis in modulating breast CSCs and its potential as a therapeutic target for cancer treatment. The researchers used knockdown and overexpression approaches in various breast cancer cell lines to demonstrate the biological function of fascin in regulating β-catenin downstream targets in a FAK-dependent manner. The pathway activation is essential for promoting the self-renewability of CSCs. Moreover, gene expression analysis in patients with breast cancer revealed association of shorter survival with co-expression of fascin and FAK/β-catenin, indicating the clinical relevance of their findings.

In contrast to canonical Wnt signaling, non-canonical Wnt/Ca^2+^ pathway induces and maintains the self-renewal efficiency of CSCs independently of β-catenin in colon cancer. This pathway can modulate CSC properties by regulating gene expression and cytoskeletal changes, impacting CSC migration and invasion (8). The implication of non-canonical Wnt signaling on tumorigenesis is complex and context dependent. The study by Sarabia-Sánchez et al. explores the role of Wnt/Ca^2+^ signaling in metastasis, anti-apoptosis and chemoresistance, as well as its implication in colorectal cancer progression. Using BRAF-driven RKO, KRAS-driven SW480 and SW620 colon cancer cell lines, the researchers demonstrate that the non-canonical Wnt/Ca^2+^ cascade plays a vital role in inducing and maintaining the self-renewal capacity of colon CSCs. These findings provide valuable insights into the complex regulatory mechanisms of non-canonical Wnt signaling and their role in colon CSCs with the potential implication for developing novel therapeutic strategies targeting this pathway.

Cancer heterogeneity is also driven by other regulatory mechanisms of stemness including zinc finger transcription factors, glycosylation of the components of signaling pathways, and microRNAs. Zinc finger proteins (ZNFs), a group of transcription factors with zinc-binding domains, regulate gene expression, cell proliferation, differentiation, metastasis, resistance and apoptosis (9). Dysregulation of ZNFs, including ZSCAN1, can contribute to tumorigenesis by promoting the survival and expansion of CSC populations within tumors, leading to tumor growth and progression (9). The article by Chu et al. examines the functional role of ZSCAN1 in regulating stemness of breast cancer, aiming to provide insights into potential therapeutic targets. The authors analyzed public datasets such as The Cancer Genome Atlas (TCGA) and the Molecular Taxonomy of Breast Cancer International Consortium (METABRIC), alongside xenografts, to demonstrate the association of ZSCAN1 with breast cancer stemness. Dysregulation of ZSCAN1 may have a significant implication in tumor progression. This study enhances understanding of the role of ZSCAN1 in regulating cancer stemness and underscores its potential as a new target for the development of anti-tumor strategies in breast cancer.

Alterations in glycosylation patterns can impact the activity, localization, stability and signaling properties of CSC markers, thereby modulating CSC stemness (10). Aberrant glycosylation in CSC markers can contribute to tumorigenesis by promoting CSC properties. Moreover, specific changes of glycosylation in CSC markers enable these cells to evade immune surveillance and promote tumor immune evasion (11). Dysregulation of glycosylation in CSCs is associated with self-renewal, tumor progression, immune evasion and drug resistance development, posing a significant challenge in cancer treatment (12). The mini review by Khan and Cabral discusses the role of altered glycosylation in CSCs and its potential as a therapeutic target for cancer treatment. Altered glycosylation is a common feature of CSC markers and signaling pathways in all cancer types. The authors review the aberrant glycosylation in CSC markers (CD44, CD133, and CD24) and cellular signaling pathways (Notch, Hedgehog, Wnt/β-catenin, and Akt) that maintain CSC properties. Understanding glycosylation differences can aid in identifying biomarkers for detecting cancer progression and developing effective treatment strategies.

Epithelial mesenchymal transition (EMT) has been associated with the acquisition of stem cell-like properties in cancer cells, including CSCs. EMT-related transcription factors, such as Snail, Slug, Twist and Zeb, suppress epithelial traits and promote expression of stemness-related genes, facilitating the maintenance and expansion of CSCs (13). EMT contributes to the aggressiveness, heterogeneity, and treatment resistance of tumors, leading to tumor progression and poor clinical outcomes (14). In breast cancer, several signaling pathways involved in metastasis lead to the establishment of CSCs at secondary sites via EMT transcription factors (15). A review by Yousefnia et al. delves into the malignant characteristics of breast CSCs, such as resistance to chemotherapy and radiotherapy, metastasis, angiogenesis, and biomarkers. The authors have also reviewed the EMT transcription-factor-regulated signaling pathways responsible for maintaining stemness phenotypes and microRNAs (miRNAs) in breast CSCs. This review discusses various CSC markers, including CD44/CD24, CD326 (EpCAM) and aldehyde dehydrogenase (ALDH), and their potential to identify CSCs. Signaling pathways like MAP kinase, PI3K/Akt/NFκB, TGF-β, hedgehog, Notch, Wnt/β-catenin and Hippo, which can be dysregulated in CSCs due to genetic and epigenetic changes, are also examined. The review emphasizes the role of upregulated miRNAs in CSCs and proposes that their downregulation could be a novel treatment strategy for breast cancer. Recent therapeutic strategies targeting CSCs are also discussed.

Increasing evidence suggests that CSCs interact with components of the tumor microenvironment (TME), such as vascular and immune cells and various cytokines, to promote drug resistance in various cancers through multiple signaling pathways (16). Therefore, it is also important to clarify the mechanisms underlying the crosstalk between CSCs and the TME for the development of targeted anti-cancer therapies. In conclusion, this special edition advances understanding of CSC signaling pathways and potential therapeutic targets. By exploring various oncogenic pathways, transcription factors and molecular markers, researchers pave the way for novel targeted therapies and personalized medicine aimed at eradicating CSCs and improving patient outcomes.

## Author contributions

JW: Writing – review & editing. AM: Writing – original draft. CS: Writing – review & editing.
